# Folate Intake and Methylenetetrahydrofolate Reductase Gene Polymorphisms as Predictive and Prognostic Biomarkers for Ovarian Cancer Risk

**DOI:** 10.3390/ijms13044009

**Published:** 2012-03-23

**Authors:** Li Zhang, Wenxin Liu, Quan Hao, Lewen Bao, Ke Wang

**Affiliations:** Department of Gynecologic Cancer, Key Laboratory of Cancer Prevention and Therapy, Tianjin Medical University Cancer Institute and Hospital, Tianjin Medical University, Tianjin 300192, China; E-Mails: wenxl1009@163.com (W.L.); quanhao1999@126.com (Q.H.); lewenbao2012@126.com (L.B.); kewjia2001@126.com (K.W.)

**Keywords:** folate, methylenetetrahydrofolate reductase gene, polymorphism, ovarian cancer risk

## Abstract

Folic acid and methylenetetrahydrofolate reductase (MTHFR) may affect the development of human cancer. However, few studies have evaluated folate intake and MTHFR in susceptibility to and prognosis of patients with ovarian cancer. We conducted a prospective case-control study in 215 ovarian cancer patients and 218 controls (all Chinese) between Jan. 2004 and Jan. 2007. MTHFR C677T genotyping was done by PCR-RFLP. All patients were followed up until Dec. 2010. We found a 2.43-fold increased risk of ovarian cancer among MTHFR 677TT carriers, and a decreased risk of ovarian cancer in individuals with high folate intake (OR = 0.54, 95% CI = 0.32–0.94). Cox regression survival analysis showed that among the ovarian cancer patients, those carrying the 677TT genotype had a higher risk of death (HR = 2.17, 95% CI = 1.20–4.79), while high folate intake was associated with a lower risk of death (HR = 0.43, 95% CI = 0.33–0.88). Moreover, MTHFR 677CC carriers with higher folate intake showed a lower risk of death from ovarian cancer (HR = 0.32, 95% CI = 0.27–0.82). In summary, high folate intake may lessen susceptibility and improve the prognosis of ovarian cancer patients, while the MTHFR 677TT genotype appears to increase ovarian cancer risk and worsen its prognosis in a Chinese population.

## 1. Introduction

Ovarian cancer is one of the most deadly gynecological cancers worldwide [1]. China has a relatively low ovarian cancer incidence of 3–5 per 10^5^ females, which is about one-fourth the rate in northern Europe [2]. The wide geographic variation in incidence rates points to the role of genetic and environmental factors in the pathogenesis of this cancer. Possible risk factors for ovarian cancer include family history, tobacco smoking, infertility, low parity, and hormone replacement therapy, while oral contraceptive use and fewer menstrual cycles are associated with decreased risk [2,3]. Deficiency of nutrients, such as vitamins and microelements, has also been associated with increased risk for ovarian cancer, whereas high fruit and vegetable intake may help prevent the disease [4].

Folate is a water-soluble vitamin naturally found in green leafy vegetables, cereals, legumes, and fruits. It plays an important role in DNA synthesis, integrity, and stability, and folate deficiency usually causes defective DNA repair and chromosomal fragile site expression, leading to chromosomal breaks and micronucleus formation. Moreover, folate plays a central role in DNA methylation [5–7]. Folate deficiency leads to ovarian cancer through two mechanisms: by inducing misincorporation of uracil into DNA, thus disrupting DNA integrity and DNA repair; and by altering DNA methylation, which can alter expression of critical tumor suppressor genes and proto-oncogenes [8–10].

Methylenetetrahydrofolate reductase (MTHFR) is a central enzyme in folate metabolism which catalyzes the reduction of 5,10-methylene-tetrahydrofolate to 5-methyltetrahydrofolate. Methionine synthase then catalyzes the reaction of 5-methyltetrahydrofolate and homocysteine to generate methionine and tetrahydrofolate. Under folate deficiency conditions, MTHFR may cause point mutations and/or chromosomal breaks, facilitate the conversion of 5,10-methylene THF to 5-methyl THF, and reduce the concentration of 5-methyl THF to decrease the conversion of homocysteine to methionine. Ultimately, MTHFR plays a role in the carcinogenesis process of DNA hypomethylation [11]. The C677T variant (Ala222Val, rs 1801133) has been associated with decreased MTHFR activity, increased homcysteine levels, and an altered folate distribution [12]. Previous studies on the association between *MTHFR* C677T polymorphism and ovarian cancer have found conflicting results [4,13].

The roles of dietary folate and *MTHFR* C677T polymorphism in ovarian cancer are unclear and there have been few studies on this relationship in Chinese populations. However, low folate intake and inactive *MTHFR* C677T are associated with improved survival of cancer patients treated with first-line fluorouracil-based chemotherapy [14–16]. Since a folate pool imbalance and impaired repair mechanisms may result in DNA instability and strand breaks, and inactive *MTHFR* C677T may accelerate the carcinogenesis process of DNA hypomethylation under folate deficiency conditions, we hypothesized that folate deficiency and inactive *MTHFR* C677T may influence both susceptibility to and progression of ovarian cancer. We tested this idea with a case-control study and a case-only cohort study in a Chinese population.

## 2. Results and Discussion

### 2.1. Results

We recruited 215 ovarian cancer patients diagnosed between Jan. 2004 and Jan. 2007 for this study. 218 Controls were randomly selected from female people who requested general health examinations in the same hospital during the same period. Controls were required to be without any history of any type of cancer and frequency matched by five-year age groups. The average ages of cases and controls were 47.2 ± 7.5 years and 47.6 ± 8.1 years, respectively (Table 1). There were no significant differences between cases and controls in age, drinking, tobacco use, menopausal status, or hormone replacement use. Ovarian cancer patients tended to have borne fewer children than had controls. Oral contraceptive use was associated with a lower risk of ovarian cancer, while ovarian cancer in first-degree relatives heightened cancer risk. Most of the cancer patients received chemotherapy; only 6.0% of patients received radiotherapy.

There was a significant difference in C677T polymorphisms between patients and controls (Table 2). Individuals with the TT genotype had an increased risk of developing ovarian cancer relative to those with the CC genotype (Adjusted OR = 2.43, 95% CI = 1.32–6.32). Individuals with high folate intake were less likely to develop ovarian cancer (OR = 0.54, 95% CI = 0.32–0.94).

**Figure 1:**
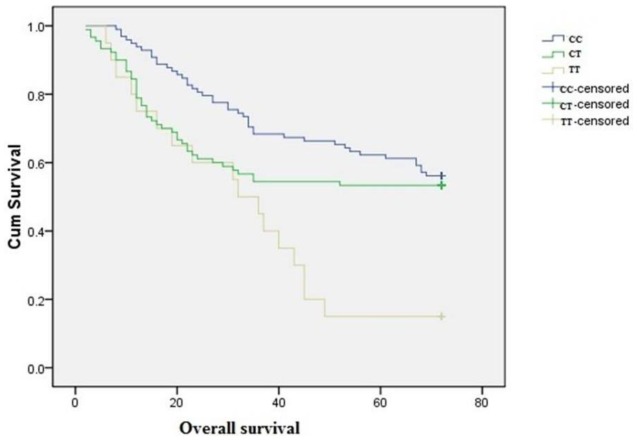
Overall ovarian cancer survival by MTHFR gene polymorphism.

**Figure 2:**
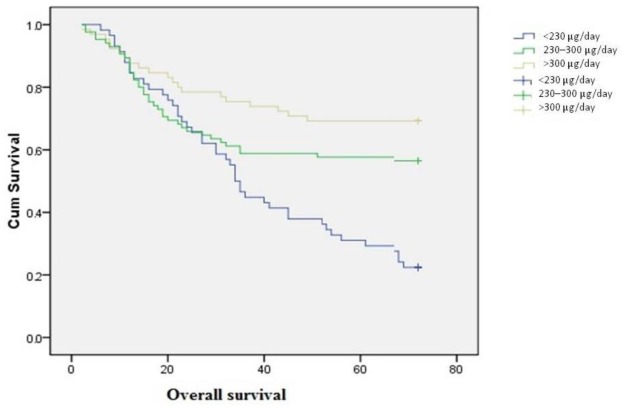
Overall ovarian cancer survival by folate intake level.

Patients were followed from diagnosis until the end of Dec. 2010. Seven of the 215 patients were lost to follow-up due to migration, while the remaining 208 patients completed the study. The median follow-up time was 48.2 months (range: 2–72 months). A total of 106 patients (50.9%) died of ovarian cancer during the follow-up period (Table 3). The 677TT genotype was associated with poor survival (HR = 2.17, 95% CI = 1.20–4.79) (Figure 1). We found an inverse relationship between folate intake and risk of death, with an average survival time of 40.5 months for individuals with low folate intake, and 56.2 months for those with the highest folate consumption (Figure 2). The HRs (95% CI) for moderate and high folate intake were 0.67 (0.40–1.03) and 0.43 (0.33–0.88), respectively (*p* for trend = 0.011).

We further explored the association between *MTHFR* C677T polymorphisms and ovarian cancer prognosis according to folate intake level (Table 4). Among individuals with a folate intake of 230–300 μg/day and >300 μg/day, *MTHFR* 677CC carriers showed a significantly lower risk of death risk from ovarian cancer, with adjusted HRs (95% CI) of 0.56 (0.39–0.96) and 0.32 (0.27–0.82), respectively. A significant interaction was found between folate consumption and *MTHFR* genotype (*p* = 0.024, data not shown).

### 2.2. Discussion

Our results indicate that *MTHFR* C677T polymorphisms are associated with susceptibility to ovarian cancer, and high folate consumption with a decreased risk of ovarian cancer. Moreover, ovarian cancer patients with high folate consumption may have better survival than individuals with low diet folate intake. This protective effect was more evident in patients carrying *MTHFR* 677CC genotypes. This may be due to the putative effect of folate metabolism on the development and prognosis of ovarian cancer.

Folate mediates the transfer of one-carbon moieties both in the synthesis of nucleotides necessary for DNA synthesis, replication, and repair, and in DNA methylation reactions [17]. These functions may play a critical role in carcinogenesis. An abundant intake of folate-rich foodstuffs appears to convey protection against developing some cancers [18]. Our study provides further evidence of the protective role of folate intake. Although previous epidemiological studies have found an inverse association between folate intake and cancer risk [19], the effect of folate supplements on carcinogenesis remains controversial for some ethnic groups [6,20]. The prevalence of variant genotypes of the *MTHFR* C677T polymorphism varies greatly among different human populations. In Africans, the frequency of *MTHFR* 677T/T is below 1%, whereas in Mexicans, it is above 30%. Its frequency in our control group was 8.8%, which is comparable to previous reports on Chinese populations [21,22]. The different results found for the effect of folate intake on ovarian cancer risk in different ethnicities may be due to differences in population background, study design, sample size, environmental factors, and chance variations. Further confirmation of such differential effects is therefore needed.

We found a significant association between *MTHFR* C677T polymorphism and ovarian cancer development and prognosis. However, previous studies have found inconsistent results for whether this association exists [13,23]. A study conducted in the United States and one Polish study both reported an association between these *MTHFR* SNPs and ovarian cancer development and prognosis [23,24], while another large sample study from the United States did not find evidence for an association between *MTHFR* C677T and ovarian cancer risk [13]. Some biological data support a role for *MTHFR* C677T polymorphism in cancer survival. Namely, the balance of the 5,10-MTHF and 5-MTHF controlled by MTHFR may influence the efficacy of chemotherapy with 5-fluorouracil (5-FU). The inactive 677TT form could increase the amount of 5,10-MTHF. Cell line experiments suggest that fluoropyrimidines like 5-FU inhibit thymidylate synthetase by forming a ternary complex involving 5-FU, thymidylate synthetase, and 5,10-MTHF [25]. Therefore, these *MTHFR* polymorphisms may enhance the effect of 5-FU by increasing the amount of 5,10-MTHF. In one animal study, administration of 5,10-MTHF enhanced the efficacy of 5-FU [26]. Another study showed that colon cancer cases with inactive *MTHFR* 677T/T are three times more likely to respond to 5-FU chemotherapy than are *MTHFR* 677C/C carriers [27]. In our study, most of the cases were treated with chemotherapy, and our results showed 677TT is associated with a higher risk of death from ovarian cancer, which indicates an interaction between *MTHFR* C677T polymorphism and chemotherapy.

Our study showed high folate intake was associated with reduced risk of ovarian cancer among 677C/C carriers, which indicates that folate and the active 677C/C genotype have a synergistic effect on ovarian cancer prognosis. This association between folate intake and reduced risk of death from ovarian cancer was only found for the 677C/C genotype. Similarly, a study conducted in China on esophageal cancer also found a significant synergy between folate intake and the 677C/C genotype [28].

Our study has several limitations. First, we selected controls from hospital visitors, which may be a threat to the validity of the results. This method brings a certain risk of selection bias, since the controls were not a random sample of the general population and may not fully represent the underlying base population. However, all control subjects in our study were those who came to the hospital for routine health examinations, not hospitalized patients with specific diseases, so they were likely representative of the general population, and thus provide a reasonable estimate of the allele frequencies of MTHFR C677T variants. Second, the small number of patients carrying the homozygous mutation TT in this study limited the statistical power of our analysis of differences among carriers of different genotypes. Increasing the number of controls to a control-case ratio of 2 or more can, at least to some extent, increase the study power. Therefore, further large-sample studies on this question are warranted. Third, collection of dietary habits information brings a risk of recall bias, which may have exaggerated or reduced the effects we found. We suggest that, for the survival analysis, future studies could collect information on dietary habits during follow-up, which would greatly reduce recall bias.

## 3. Experimental Section

### 3.1. Study Subjects

This was a case-control and case-only cohort study conducted in the Tianjin Medical University Cancer Institute and Hospital. All Chinese female cases newly diagnosed with primary ovarian cancer in the hospital between Jan. 2004 and Jan. 2007 were invited to face-to-face interviews within two months after diagnosis. All cases recruited in this study were histologically confirmed. A total of 215 cases were selected and interviewed. Controls were randomly selected from people who requested general health examinations in the same hospital during the same period. Controls had no history of any type of cancer, and were frequency-matched by 5-year age groups. Among a total of 246 eligible controls, 218 were successfully interviewed and donated blood samples, with a participation rate of 88.6%. All the patients were followed up until Dec. 2010, and we recorded all deaths of patients.

### 3.2. Data Collection

A face-to-face interview was conducted with a structured questionnaire to collect information on demographic factors and clinical characteristics, menstrual and reproductive history, hormone use, dietary habits, tobacco and alcohol habits, and family history of ovarian cancer. The tobacco smoking status were divided into smokers (smokers were defined as individuals who smoked more than 20 packets of cigarettes per year, or smoked more than one cigarette per day and continued for 6 months) and nonsmokers, drinking status were divided into drinkers (defined as individuals who drank more than 100 g alcohol (400 mL beers, 250 wine mL and 100 mL white spirit) per month and continued for 6 months) and non-drinkers. Information on usual dietary intake was collected using a comprehensive quantitative food-frequency questionnaire (FFQ) with 95 items. The FFQ used in this study was based on a questionnaire designed by the US National Institutes of Health and modified according to common Chinese food items and cooking habits. For each food item, we collected the frequency and quantity of consumption, then calculated daily intake by multiplying the reported consumption frequency for each food item by the portion size specified by the respondent. Soy food intake was estimated with 16 soy food items, including tofu, soy milk, soy yogurt, soy frozen yogurt, soy ice cream, soy cheese, soy hot dogs and cold cuts, other meat substitutes made from soy, tempeh, miso, soybeans, roasted soy nuts, soy sauce, soybean sprouts, alfalfa sprouts, and protein powder supplements made from soy.

The study protocol was approved by the Ethics Committee of the Affiliated Hospital of Sichuan University. Informed consent was obtained before each interview.

### 3.3. Genotyping

Genomic DNA was extracted from whole-blood samples using the Qiagen Blood Kit (Qiagen, Chatsworth, CA). Genotyping was conducted by TaqMan assays using the ABI Prism 7911HT Sequence Detection System (Applied Biosystems, Foster City, CA). Primer, probes, and reaction conditions were based on those of our previous study [29]. Genotyping was done by laboratory personnel blinded to case-control status. For quality control purposes, we also genotyped internal positive control samples, used no-template controls, and replicated the genotyping of 10% of the samples.

### 3.4. Statistical Analysis

All statistical analyses were performed using STATA 10.0 (College Station, TX, USA). Differences in demographic and clinic characteristics, as well as potential confounding factors, were tested using a chi-squared test. Previous studies showed that family history of ovarian cancer, reproductive factors, menopausal status and hormone replacement therapy were the risk factors for ovarian cancer [30], and these factors may confound the association between MTHFR C677T polymorphism and ovarian cancer prognosis. Moreover, previous studies showed that there was interaction between MTHFR gene polymorphism and tobacco smoking and alcohol consumption [31–33]. Therefore, we adjusted the age, tobacco smoking, alcohol consumption, number of deliveries, menopausal status, hormone replacement, oral contraceptive use, and ovarian cancer history when analyzing the association between MTHFR C677T polymorphism and ovarian cancer prognosis. Unconditional logistic regression was undertaken to estimate odds ratios (ORs) and their 95% confidence intervals (95% CIs) after controlling for potential confounding factors. In the logistic regression equation, the age was continuous variable, and tobacco smoking, alcohol consumption, number of deliveries, menopausal status, hormone replacement, oral contraceptive use, and ovarian cancer history were categorical variables.

The outcome measured was overall survival, which was estimated using the Kaplan-Meir method. A univariate Cox regression analysis was used to assess the association between folate intake, MTHFR C677T*-Arg399Gln* gene polymorphisms, and survival. A primary death from ovarian cancer was defined as a failure event, and the survival time was defined as the time between diagnosis and death. The cause of death was defined by specialists based on clinical documents and reports by patients’ family members. If a patient died from a cause other than ovarian cancer, her data was censored at the date of death. All surviving patients were censored at the date of last follow-up. Folate intake was computed by multiplying food intake (in grams) by folate content (per gram) of the food, then summing all folate intake from various foods to obtain the total folate intake. The continuous variable of folate intake was categorized as low, moderate, or high using tertiles as cut-off points. The relative risk [hazard ratio (HR)] and 95% CI were calculated with the Cox regression model for all significant predictors from cancer diagnosis to the endpoint of the study (event). All statistical tests were two sided, and differences were taken as significant when the *P* value was less than 0.05.

## 4. Conclusions

In this first study to investigate the association among folate intake, *MTHFR* C677T polymorphism, and ovarian cancer susceptibility and survival in a Chinese population, we found that high folate intake may protect against the development and improve the prognosis of ovarian cancer. The 677TT genotype appears to be positively associated with ovarian cancer risk and poor disease prognosis. Further studies with larger sample sizes are needed in Chinese populations.

## Figures and Tables

**Table 1:** Demographic and clinical characteristics of ovarian cancer patients.

Variable	Cases, *N* (%)	Controls, *N* (%)	*p* value
**Age, years [mean, (sd)]**	47.2, 7.5	47.6, 8.1	0.30
**Smoking status**			
Smokers	196 (91.2)	204 (93.5)	0.34
Nonsmokers	19 (8.8)	14 (6.5)	
**Drinking status**			
Drinkers	169 (21.6)	177 (18.8)	0.50
Nondrinkers	46 (78.4)	41 (81.2)	
**Number of deliveries**			
0	27 (12.6)	12 (5.7)	<0.05
1	85 (39.6)	79 (36.4)	
2	83 (38.7)	95 (43.7)	
≥3	20 (9.1)	31 (14.2)	
**Menopausal status**			
Pre-menopausal	105 (48.7)	97 (44.3)	0.37
Post-menopausal	110 (51.3)	121 (55.7)	
**Hormone replacement therapy**			
Never	203 (94.5)	212 (97.2)	0.14
Ever	12 (5.5)	6 (2.8)	
**Oral contraceptive use**			
Never	162 (75.3)	140 (64.4)	<0.05
Ever	53 (24.7)	78 (35.6)	
**Ovarian cancer in first-degree relatives**			
Yes	203 (94.4)	217 (99.994)	<0.05
No	12 (5.6)	1 (0.006)	
**Tumor type**			
Invasive	132 (61.6)		
Borderline	81 (37.5)		
Unknown	2 (0.9)		
**Chemotherapy**			
Yes	154 (71.6)		
No	44 (20.5)		
**Radiotherapy**			
Yes	13 (6.0)		
No	185 (86.0)		

**Table 2:** Frequency distribution and association of methylenetetrahydrofolate reductase (*MTHFR*) C677T genotypes and folate intake levels with ovarian cancer.

Genotype/Allele	Cases, *N* (%)	Controls, *N* (%)	OR 1 (95% CI)	OR 2 (95% CI)
MTHFR C677T				
CC	102 (47.3)	115 (52.8)	1.0 (Reference)	1.0 (Reference)
CT	94 (43.9)	92 (42.1)	1.15 (0.76–1.74)	1.48 (0.94–2.15)
TT	19 (8.8)	11 (5.1)	1.94 (0.83–4.75)	2.43 (1.32–6.32)
T allele	113 (52.7)	103 (47.2)	1.24 (0.83–1.84)	1.67 (0.99–3.27)
**Daily folate consumption (**μg/day**)**				
Mean (SE)	257.4, 36.7	295.5, 28.6	-	
<200	61 (28.6)	46 (21.3)	1.0 (Reference)	1.0 (Reference)
200–310	90 (41.7)	95 (43.7)	0.71 (0.43–1.19)	0.66 (0.41–1.05)
>310	64 (29.7)	76 (35)	0.64 (0.37–1.09)	0.54 (0.32–0.94)

1 None adjusted OR.

2 Adjusted for age, tobacco smoking, alcohol consumption, number of deliveries, menopausal status, hormone replacement, oral contraceptive use, and ovarian cancer history.

**Table 3:** Kaplan-Meier survival estimation of median survival and HRs with MTHFR C677T gene polymorphism.

	*N* (%)	Mean Survival, 95% CI (months)	HR (95% CI), *p* value
MTHFR C677T			
CC	98 (47.2)	54.5 (69.9–59.1)	1.0 (reference)
CT	90 (43.1)	46.0 (40.1–51.9)	1.34 (0.89–2.18), 0.11
TT	20 (9.7)	33.8 (24.6–43.0)	2.17 (1.20–4.79), <0.05
Folate intake (μg/day)			
<230	58 (27.9)	40.5 (34.5–46.5)	1.0 (reference)
230–300	88 (42.2)	48.8 (42.9–54.7)	0.67 (0.40–1.03), 0.06
>300	62 (29.9)	56.2 (50.2–62.3)	0.43 (0.33–0.88), <0.05

**Table 4:** MTHFR C677T polymorphisms and ovarian cancer prognosis according to folate intake level.

Daily folate consumption	Genotype								

	CC Cases (%)	Deaths *N* (%)	HR (95% CI) 1	CT Cases	Deaths *N* (%)	HR (95% CI) 1	TT Cases	Deaths *N* (%)	HR (95% CI) 1
<230 μg/day	30 (30.6)	23 (51.1)	1.0 (Reference)	20 (22.2)	16 (38.1)	1.0 (Reference)	8 (40.0)	6 (40.0)	1.0 (Reference)
230–300 μg/day	33 (33.7)	14 (31.1)	0.56 (0.39–0.96)	48 (53.3)	18 (42.9)	0.59 (0.42–1.27)	7 (35.0)	5 (33.3)	1.25 (0.84–1.45)
>300 μg/day	35 (35.7)	8 (17.8)	0.32 (0.27–0.82)	22 (24.5)	8 (19.0)	0.45 (0.34–1.02)	5 (25.0)	4 (26.7)	1.59 (0.93–2.24)

1 Adjusted for age, tobacco smoking, alcohol consumption, number of deliveries, menopausal status, hormone replacement, oral contraceptive use, and ovarian cancer history.
